# An Inverted Container in Containing and Not Containing Hospitalized Patients—A Multidisciplinary Narrative Inquiry

**DOI:** 10.3389/fpubh.2022.919516

**Published:** 2022-07-08

**Authors:** Gillie Gabay, Smadar Ben-Asher

**Affiliations:** ^1^Multi-Disciplinary Studies, Achva Academic College, Shikmim, Israel; ^2^Educational Psychology, Achva Academic College, Shikmim, Israel

**Keywords:** narrative, acute care, clinicians, hospitalization, containing, patient-centered care

## Abstract

**Objective:**

Patient-centered care calls to contain patients in their time of crisis. This study extends the knowledge of provider patient interactions in the hectic environment of acute care applying Bion's container-contained framework from psychoanalysis.

**Methods:**

Following ethical approval, we performed a narrative inquiry of the experiences of ten patients upon discharge from lengthy hospitalizations in acute care. Interviews were conducted upon discharge and about one-month post-discharge.

**Findings:**

Data analysis suggests four modes of containing of patients by providers. In nurturing interactions, typical of an active container-contained mode, patients experienced humanized care, symptom control, hope, and internal locus of control. This mode yielded patient gratitude toward providers, wellbeing, and post-discharge self-management of diseases. In rigid and wall-free modes of containing, patients experienced a sense of powerlessness and discomfort. A new mode of container-contained was identified, the “Inverted Container”, which extends Bion's theory and contradicts patient-centered care. In inverted containers, patients contained the providers yet reported feeling gratitude toward providers. The gratitude constitutes a defense mechanism and reflects a traumatic experience during hospitalization, which led to post-discharge distrust in providers and hospitals and poor self-management of illness.

**Conclusions:**

To effectively provide patient-centered care, provider-patient interaction in lengthy hospitalizations must move along a clinical axis and a relationship axis. This shifting may facilitate containing patients in their time of crisis so essential processes of reflection, projection, and transference are facilitated in-hospital care.

## Introduction

The Institute of Medicine (IOM) defined patient-centered care (PCC) as one of the six fundamental aims of health care systems (1, 2). PCC is care that establishes a patient-provider partnership; ensures respect for patients' needs, and preferences; assures that patients have the required literacy to make decisions; and supports patient involvement (3, 4). The key dimensions of PCC are (a) Respect for patient values, preferences, and needs; (b) Coordination and integration of care, information, communication, and education; (c) Physical comfort, entailing pain management, assistance in daily living, and comfortable surroundings; (d) Emotional support and alleviation of fear and anxiety; (e) Involvement of family; (f) Transition and continuity of care; and (g) Access to care (5, 6). Orienting care around patients' needs improved patients' clinical outcomes, reduced both under-utilization and overutilization of health services, and enhanced satisfaction of patients and providers alike (7). One influential model underlying PCC is the Planetree Model (8) that explicitly recognizes the importance of human interaction in medical care. It views effective interactions as nurturing interactions, encompassing kindness, presence, and empowerment of patients from diverse backgrounds (8, 9). PCC places value on the individual's personhood and autonomy, including patient's wishes regarding their healthcare (10).

Research demonstrated that nurturing patient-provider interactions shape the quality of care (2, 11). Nurturing interactions of providers with patients require inner resources and a human touch (6). PCC requires providers to understand patients' biopsychosocial context, ensure patient understanding of the clinical condition, and share power and responsibility (4, 11, 12). PCC emphasizes an egalitarian relationship between patients and providers with the recognition that power asymmetries can be detrimental to patients, particularly to those whose complaints are dismissed or disputed, and for those lacking knowledge and skills to facilitate communication with providers (11, 13).

Despite the essential importance of PCC to higher quality of care, and despite the growing evidence regarding its importance to patients, providers, and health systems, hospitals are far from achieving PCC (6, 11, 14–17). Previous studies theorized the benefits of nurturing provider–patient interactions, and other studies tested the implementation of PCC in community settings, but studies that elucidate the perspective of patients and their explicit expectations of providers in acute care are scant (2–4). It is important to examine how providers implement PCC in practice in acute care. This study fills a gap in the state-of-the-art, borrowing from psychoanalysis to medicine to explore patterns of provider-patient interactions from the perspective of patients who underwent lengthy hospitalizations in acute care. We draw on the theoretical framework of Bion's container-contained theory.

## Bion's Container-Contained Theory

The central concept of the 'container' in psychoanalysis, as formulated by Bion (18), relates to a helper accepting the needy and their needs, an initial emotional contact that is a critical dimension in every interaction. Bion's conceptualization of the container in relationships emerged from psychoanalysis, originating in the description of patterns of responses by a mother to her baby's needs. Since patients completely depend on their providers, similarity in characteristics to those of the mother and baby enable us to apply Bion's theory to relationships in medical encounters focusing on the development and growth of patients whose providers encourage and empower them in healing processes. Bion (18, 19) described the mother as a container that provides the baby an emotional presence without words, through attention to the baby who is unable to express itself in words. The mother perceives signals from the helpless baby, picks up the messages and names them. This broadcast-translation function is the most important containment function (Appendix—Exhibit A elaborates on this broadcast translation function and identification mechanisms). Bion (18) presented three modes of 'container-contained' which differ in the extent to which the psychotherapist, in short-term interventions, contains the client, allowing them to reflect and process emotional stressors through a transformative process. Incapacity to contain constrains such transformations.

The first mode of container-contained is the active container, which contains the client, allowing psychodynamics to take place. The second mode is a container that inconsistently contains the client, and the third is a rigid container, which rejects information from the client (verbal and non-verbal), as though fully blocking the dynamic between the subject and the object. Since Bion (18) focused on relationships with asymmetrical power, such as the relationship between mother and baby, it is appropriate to apply this theory in exploring asymmetrical relationships of power between providers and patients in acute care. We argue that in a provider-patient relationship, the provider's emotional capacity, not necessarily verbal, can signal an understanding of the patient's distress and mitigate anxiety and fear (12). The provider may translate the patient's pain, fear, and distress into words and provide the patient with hope for a good future where the patient may develop resilience, cope with the pain, and manage the illness (4). The provider may instill hope for improving the patients' health condition. In the patient-relationships, the provider's emotional presence is of great significance (20).

Applying Bion's theory (19) to address the uniqueness of each patient, the provider, as a container, must be free to absorb the patient's unique experience. The inner container is an expression of the type of ability that varies from person to person, associated with curiosity and learning from experience. It is not related to an interpersonal dimension but expresses the ability to look at the world as a source of knowledge (18). The words give meaning to emotions, clarify and sooth. The theoretical understanding of projective identification enables us to examine the provider-patient relationship in situations where mental content is transmitted to the provider, evoking anxiety or a feeling of disintegration in patients (21–23).

The provider can absorb this content as a temporary container, holding the unbearable inner experience of the patient until the patient can deal with the emotions evoked. When providers manage to absorb the patient's anxiety and hold it empathetically for that patient, they serve as a “container-container”. These container-container relationships may constantly evolve and grow in an ongoing process of mutual influence that allows for the transformation of thoughts and feelings. A second condition is when providers hear the patient's complaints but are unable to hold the experiences, thoughts, and anxieties and collect them for the patient, thus, acting as a “rigid container” (24). The rigid container does not allow any expression of contents to seep into it. The container seems to refuse to comment on what was inserted into it. The third mode describes providers who do not perceive the patient's distress at all act and as a “container without walls”. This state is present when the content conveyed in the process of projective identification is loaded turbulently and has “explosive” qualities. The provider is unable to contain and hold the content for the patient. In such cases the provider-patient relationship is fragile, and the main experience of the patient is that of lack of capabilities. Since we argue that there is a similarity between the mother-baby's interaction and that of provider-patient, we examined the theoretical principles presented by Bowen in this relationship.

In the present study we borrow Bion's (18, 19) theory of container-contained from psychotherapy to provider-patient relationships in lengthy hospitalizations, seeking to examine the modes of containers in interactions between providers and patients in lengthy acute care hospitalizations. In hospitalizations, patients expect providers to contain their pain, understand it, and acknowledge their crisis (4). Having their expectations met may alleviate their anxieties and concerns. The provider who contains emotions and thoughts of patients contribute to understandings of patient's processes, emotions, and thoughts. When patients' expectations are unmet by providers, there is no containing. If the patient can contain the frustration, it may facilitate growth (4, 12, 20). The current study, examining the provider-patient interaction, aims at identifying the container-contained modes that providers use and their relation to attaining PCC. There is a paucity of literature discussing the modes of container-contained in provider-patient relationships in lengthy acute care hospitalizations. The research questions are: (1) How did patients experience the provider as a container during their lengthy hospitalization in acute care? (2) What conduct of providers characterizes each mode of container-contained? (3) How does each mode of containing promote or undermine PCC?

## Methods

### Recruiting Participants

We employed a maximum variation approach in recruiting participants to include a wide range of perspectives (25). Participants were 10 Jewish secular Israelis (six men and four women), ages 29 to 81, with diversity in participants' age, gender, geography, illnesses, profession, and work status. Participants were hospitalized in a large hospital (1,202–3,200 beds) or medium hospital (300–700 beds). Sample size was determined by the information saturation method (26). Table 1 presents demographics and health attributes by group (27). Participants were hospitalized due to cancer, heart disease, neurological disorders, or life-threatening accidents.

**Table 1:** Sociodemographic data by group, type of disease and profession.

**Age group**	**Disease**	**Profession and status**	**No. of Children**
Young (319–36); Three participants	Spine Cancer; Uterus Cancer; Neurological; Crushed both arms and hands	Software, employed; Engineer (self- employed); Dancer (self-employed); Designer (self-employed)	0–2
Middle (42–58); Four participants	Breast cancer; Upper limbs injury; Lung cancer	Teacher (employed); Architect (self- employed); Photographer (employed)	2–4
Older (66–81); Five participants	Sternum cancer; Neurological; Uterus Cancer; Heart	Consultant (self- employed); Insurance (employed); Psychotherapist (self-employed); Retired	2–4

A snowball sampling was used to locate subjects during their initial recovery process upon discharge from an acute-care setting in a public general hospital. Participants were hospitalized for about 3 weeks. Interviews were audio-taped, transcribed verbatim, and translated from Hebrew to English.

### Procedures

Ethical approval was granted (IRB #099, September 2017). Following ethics approval, participants were recruited. The first author assured participants that their participation would have no influence on their future treatments at the hospitals and informed them that they could stop the interview whenever they choose. She asked participants to sign a written statement of informed consent regarding participation in the study and publication. Participants acknowledged their understanding that parts of their narrative will be published (27). All identifying demographics of individuals were omitted from the Findings section to ensure anonymity and confidentiality (27). All names used are pseudonyms. After transcribing the interviews, each participant received a copy and approved their content. Two participants asked to omit a paragraph from the interview due to risk of disclosure. The first author presented herself as a researcher from academia studying the hospitalization experience. She presented the goal of the study as education and improvement based on patients' experiences. She also presented the study methodology. A total of 20 interviews were conducted, two interviews for each participant to share the hospitalization experience with the interviewer. The first author conducted the interviews at participants' homes upon discharge during the initial phase of recovery. The first interview was conducted within the first 2 days after discharge, and the second interview about a month after. Interviews ranged in length from 90 min to 2 h; two interviews lasted 3 h and one interview lasted 4, due to considerations of physical discomfort or emotional distress that required breaks. Participants stressed that although it was very challenging for them to meet so soon upon discharge, they had a purpose, i.e., to improve the experience for others.

As in narrative interviews, the first author asked one general, open-ended question aimed at generating a deep, unstructured narrative (28): “Please tell me, how you arrived at the hospital and what did you experience there?” From then on, participants shared their experience from the first appearance of symptoms until discharge. The interviewer listened actively and made no attempt to comment on, ask questions, or judge what participants said. To allow participants to express themselves freely throughout the emotional interviews, the first author endeavored to have her body language send a message of acceptance even when it was emotionally difficult to contain the narratives (29).

### Research Quality Criteria

We maintained general quality standards of qualitative research (25). We acknowledged our own theoretical positions and values regarding the research issue; we acknowledged our shared experience with participants requiring examination of our critical reflections from hospitalizations (30), and our privileged position as academic researchers and as native Hebrew-speakers from the same culture. To support the transferability of the findings, we described the methodology of the study in detail and provided dense descriptions of participants' points of view. Narratives were told in upon discharge and a month later during the initial phase of healing, in the patients' homes. Narratives were anchored within three contexts that affected the participant's choice of the narrative the broad context, the micro-context, and the immediate context (28). The broad context of the narratives was the Israeli universal health care system providing all residents broad health services (31). Similarly, to other health systems in the Organization for Economic Co-operation and Development (OECD), the Israeli health system exposes its patients and providers to difficulties of shortages in resources (32). There is a shortage of providers and a decreasing rate of beds per population (33). In 2013, the Israeli Health Ministry instructed hospitals to adopt PCC as the cornerstone of quality health care. The micro-context was each participant's stage of life and career. Finally, the immediate context of the “here and now” may have also affected the narrative: the way the first author defined the study, her academic identity as an audience for their story, and participants' wish to participate in the study. Thus, the narrative is the story each participant chose to tell rather than everything that happened during the hospitalization (34).

### Data Analysis

Thematic analysis was guided by Saldana (35), aimed at exploring patient experiences in the relationships with providers and the mode of container: active, containing, or rigid. We identified themes, i.e., units derived from patterns such as recurring meanings and feelings, by bringing together elements of ideas or experiences, which often, when viewed alone, are meaningless but make sense in a specific context (35). Themes emerged from the data through six analytical steps: (1) We independently read and re-read the interviews and listed patterns of experiences through direct quotes. (2) We then identified all data that related to the patterns already classified. (3) We sorted all data according to the corresponding pattern. (4) We combined and categorized related patterns into sub-themes to obtain a comprehensive view of the emerging modes of container contained. (5) We pieced together themes in a meaningful way to form a comprehensive picture representing the patient's interpretation of their collective experience of their relationships with providers (35). (6) By referring back to the literature, we obtained information that allowed us to make inferences from the data regarding the provider-patient relationship and the mode of container-contained.

## Findings

In this section we present the testimonies of patients describing their personal encounter with physicians. We present the analysis of all the interactions that characterize and demonstrate each of the three containers in the provider-patient dynamic and examples of a fourth mode, the inverted container where the patient is concerned with the wellbeing of the provider.

### The Active Container

The physician perceives the patient's distress as an active container and feels responsible not only for the technical aspects of the treatment but also for the person themself. This physician seeks a personal connection, is interested, listens, and tries to solve problems. The patient feels free to communicate themselves, their fears, and loneliness, creating a sense of togetherness. The experience of caring empathy and shared time enables increased confidence and affects the patient's ability to cope with the disease.

“*I was happy when the anesthetist came and introduced himself, told me what would happen, and how I would feel” (Ella, 66); I remember the moments with the doctors along all the winding, tangled roads. They were concerned, attentive, and I received much support. Their benevolent presence encouraged me*. (Michelle, 74).

One of the significant components of the container is the personal care that provides the patient with their unique identity and offers hope:

“*A tiny doctor with hair standing-on-end came into the room, hugged me, and said: You will not die on me.” (Koby, 52)*.

The patient and the doctor sometimes have a close relationship:

“*I talked to her about things I could not talk to anyone.” “She (the oncologist) was sitting with me, talking philosophy, film, literature, music, I connected with her on a very intimate level, that's how she maintains relationships with her patients”* (Koby, 52)

While providing care it is sometimes necessary to choose among treatment options. Involving the patients in decision making is part of creating trust and acknowledging their possible contribution to making the right decision:

“*Then she asked me what I do in life.” (*Ella, 66); “*The doctor treated me like a person, asked about my children.”* (Daniel, 35)

What is special about these testimonies is the acknowledgment of the patient as a person who has another life outside the hospital, and the disease is not the person but rather an event in the person's life. The emotional presence is not necessarily verbal. Eye contact, attention, and stopping at the patient's bedside and checking the treatment they are receiving all create an ongoing experience of a benevolent presence.

“*My doctor and I became emotionally connected. She started at 7 in the morning, arriving with coffee, chocolate and would stay after work*.” (Koby, 52); “*The department director would see me in the hallway and call out: “It's going to take you a while but everything's fine. He always asked how I was, even when he saw me from a distance, although he did not treat me, it was important for him to encourage me* (Martin, 62).

The provider was perceived as helping and assisting both medically and emotionally:

“*She said I can always spin the rules of the game to get the best out of it, it helped me overcome my fears, get back to life as whole as possible, she strengthened me so I could deal with the disease. (Martin, 62); “I shared with the doctor what I was going through, and especially the anger and sadness and my resistance to accept my new medical reality, I was very upset. She listened, and then said to me, “I thought about what you said and told me.”* (Michelle, 74)

In the patient-provider relationship, there is a common tendency to attribute to providers the powers of a savior or angels. This de-personalizes and distances providers from everyday human reality:

“*The team was really a legend”* (Joel, 81); *They are angels doing sacred work there*.(Michelle, 74*)*.

### The Rigid Container

When the doctors hear the patient's complaints but continue to follow regular procedures without changing or empathetically containing the pain, we describe it as a rigid container mod*e:*

“*I shouted...” Nurse….Nurse….. Finally the nurse came with a paper pot, turned on the light, waking all the other patients. Only much later did she return to take the pot. When I needed to pee again, she brought me the same pot. The pot collapsed beneath me and I lay in a puddle of urine.” (*Ella, 62)

It seems that the nurse was working according to procedures but without relating to the patients to whom she is delivering care. Patients describe esteemed professionals as doing a very good job clinically but not “seeing” the patient:

“*He is a professional.”; “The surgeon is very matter-of-fact but does not accompany you, he is like a technician who comes to repair the damage”; The hospital is an industrial plant, and I am the kettle that needs to be fixed.“* (Ella, 66*); ”The nurses are technocratic, they do not talk with you, they do their job and that's it“* (Mike, 35)*; ”I would ask providers to remember that the patient is a person just like them, with feelings, scared, sad, weak, exposed, vulnerable, just like them. All they have to do is say “hello”, smile, ask how you feel... Overall, it takes a few minutes with each patient.“* (Ella, 66)

Fear is the main emotion that patients report. One patient explains the distance that doctors create from patients as a defense mechanism that prevents their encounter with patients' feelings of fear. They assess their experience of contact with the doctor according to the doctor's ability to alleviate fear:

“*Maybe he spoke like a professional doctor, but I understand the words and it shocked me. I was terrified. I thought he would talk to me about what symptoms I have, explain to me if the symptoms are normative so I wouldn't be so anxious. But he didn't even share the test results with me.”* (Jacob,78)

In the rigid container, patients report that they experience the connection with the doctor as satisfying their physical needs without actively absorbing their difficult feelings and helping process them.

### A Wall-Free Container

This mode describes the provider as failing to meet patient's needs. For patients the phenomenon mostly described the provider as ignoring them:

“*The two doctors talked about the medical procedures I have to go through as a routine arrangement, like arranging flowers.... I wondered if they see me at all. Do they know it's my cancer?”* (Ella, 66*); “The doctors just don't hear anymore.”* (Joel, 81)

The main experience is ignoring them:

“*I don't think the doctor came to visit me after the surgery” (*Jacob, 78*); “No one spoke to me. Not a surgeon, not a social worker, not a psychologist. The feeling of loneliness was difficult.”* (Ella, 66)

The patient's difficulty lies in not being seen as a suffering person*:*

“*The young doctor in the emergency room did not take my complaints of excruciating pain seriously (Daniel, 35);” When we got to the emergency room this time, there was the same doctor again. I had such strong pains, and he didn't understand the intensity of the pain at all. Sent me back to the community for testing. … I think that he recognized me. He dumped me like a dog “Go do an abdominal, liver, kidney ultrasound.” (Joel, 81); “There is a lot of loneliness”. (Ron, 60); The whole conversation between them was over my head. Gathering around the bed talking about you like you're a piece of meat” (Jacob, 78); “All the doctors talked about me like I'm not in the room. Like I'm not there … not a person… a museum exhibit.. just another object.”* (Ella, 66)

Some patients experience the lack of humane treatment as abuse. One patient asked for answers and the doctor ignored his request. When the patient complained to the nurse, she replied that she is not the doctor's lawyer. Another patient recounts a medical abuse when he was helpless:

*I woke up one night in the hospital with stab wounds in the hand. I opened my eyes to see a young woman with a needle trying to jab me in the hand.. she said she was practicing... I was drugged and unable to complain.” (Koby, 52); “They didn't listen to me even though I told them I was sensitive to iodine” (Gigi, 29)*.

The data analysis revealed a fourth container mode, an inverted container, where the patient is the one who contains the provider's distress.

### The Inverted Container

The inverted container is where the patient attends to the provider's distress and is preoccupied with it. In this situation the patient assumes the role of the ”good patient“, avoids reporting pains to the provider to avoid giving further burden. Some of the participants presented attitudes of empathy toward providers in distress*:*

“*They have insane workloads….“their living conditions are unbearable” (Dalia,72); “They are so burned out, they are missing nurses, missing technicians, they are completely dehydrated* (Ron, 60*)*.

The doctors and nurses become the object of concern; they must take care of their wellbeing so as to able to continue their medical work:

“*The morning before I was discharged, my mother asked the nurse, “How are you?”... and the nurse answered, “Well, it's nice someone is asking how I feel”…...She was so bitter“ (Daniel, 35); I do what I can to help, sometimes I closed my eyes. All the horrible sights you see there.”* (Martin, 62)

Patients themselves become preoccupied with their doctors' experience, their physical and mental difficulties, their employment conditions, their fatigue, and also their knowledge limitations:

“*Doctors shoot a lot in the dark, their motives are altruistic, and they try to help as much as they can, but their knowledge is limited. They carry insane burdens*.” (Gigi, 29).

In a state of inverted containers, the patient develops feelings of pity for the doctors and a desire to help them:

“*I felt sorry for the doctors who work so hard. It is difficult to survive as a doctor in a hospital. The doctor cannot help due to heavy workloads and not enough resources” (*Ella, 66); *The doctors are too tired... exhausted. They have a hard time making a living*. (Michelle, 74)

**Figure 1:**
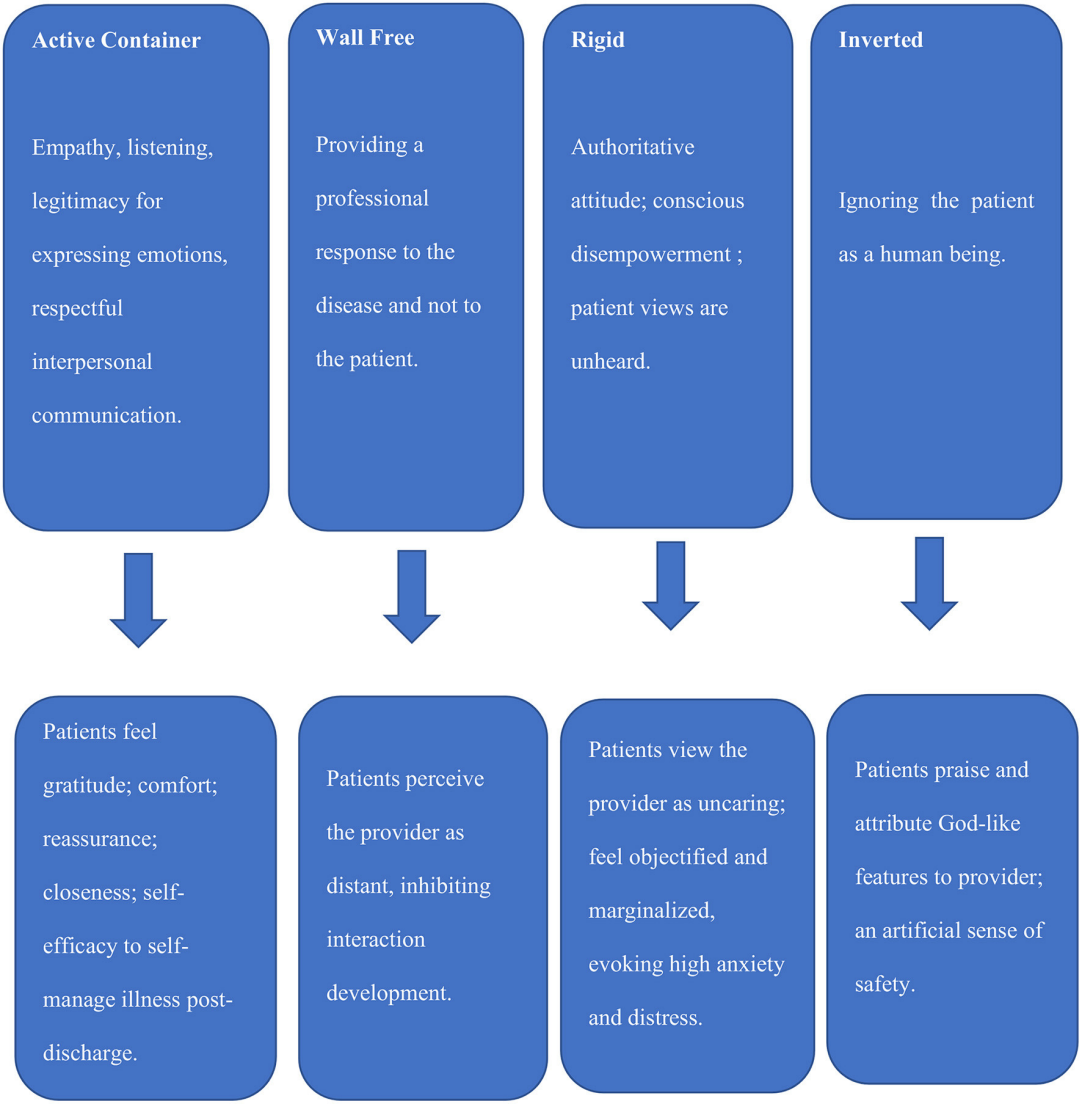
Modes of containing and not containing in patient-provider interactions and their impact on PCC implementation.

Patients engaged in receiving physicians' distress signals are deeply anxious because the physician's weakness threatens their chances of receiving proper care, thus, being empathetic to the provider may mitigate their anxiety. Figure 1 presents the four Modes of Container and Implementation or Undermining of PCC in the provider-patient interaction.

## Discussion

This study borrowed Bion's (18) container-contained theory from psychoanalysis to medicine to explore container-contained modes in provider-patient interactions in lengthy acute care hospitalizations, based on patient narratives within the context of PCC. This study makes several contributions. Theoretically, the study elucidated providers' conduct in provider-patient interactions, extending the knowledge on nurturing interactions in the hectic environment of acute care. Applying the container-contained framework, we identified an emerging fourth mode of container-contained, the 'Inverted Container'. Methodologically, this study is a rare investigation of patients' experiences and perspectives upon discharge from a lengthy hospitalization and again, about a month post-discharge. Practically, this study makes recommendations for developing active modes of container-contained in the delivery of hospital care.

### Container-Contained Modes in Provider's Conduct in Acute Care and Patient Experience

Four modes of containing were identified. Participants who experienced nurturing interactions, typical of an active container-contained mode, expressed gratitude toward providers and reported higher wellbeing. Participants experienced comfort through humanized care, symptom control, hope, and internal focus of control (4, 20, 36, 37). Discomfort was caused by participants' sense of loss and powerlessness. The inverted container in which the patients contained the providers depicts a defense mechanism by patients who experienced traumatic or poor relationships with providers, breaching their trust in physicians and in the hospitals, and negatively affecting self-management of illness post-discharge, all contradicting the moral imperative and the PCC approach. Rigid containers and inverted containers distance hospitals from capacity to achieve PCC. Below the provider's conduct in each of the four modes of containers.

### Conduct in Active Container-Contained Mode

Four participants experienced being nurtured when providers attempted to reduce their distress by taking the time to talk with them patiently, to explain things, and legitimize their emotional experience (37, 38). Participants expressed gratitude when providers enabled them to talk about their fears, anxieties, and problems, alleviating their anxieties (38). Participants felt that providers offered them comfort and reassurance when they provided bedside presence before procedures, creating the opportunity for intimate, private communication, and closeness that enabled participants to ask more questions and share their concerns and feelings (20, 37). This private time empowered participants, showing that the provider believes in their ability to overcome the temporary distress and to improve self-management of illness. They felt the provider as very involved in their medical situation, providing a sense of safety and security that alleviated negative thoughts and emotions. Providers demonstrated respect for patients' values and preferences by active listening to their concerns, providing information, relieving participants' distress, and encouraging them to express their emotions. Providers reflected on room for improvement of patients' conditions and enhanced patients' perceived control of the situation, which was found to promote self-management of illness and quality of life post-discharge (4, 36, 37, 39). Such a provider-patient nurturing relationship assisted participants in the face of emotional difficulties in their time of crisis due to the hospitalization or readmission, in cases of a progressive disease or acute conditions (37). Participants needed to feel the provider's attention, empathy, and acknowledgment of their crisis, facilitating wellbeing and growth (4, 20).

### Conduct in a Container-Contained Wall-Free Mode

Lack of nurturing in provider-patient relationships made three participants feel that providers focus only on their physiological conditions and on treatment, disregarding the “person inside” and their distress. Participants longed for providers' presence as a reassurance that they will get well. Some participants perceived the provider as distancing from them. A distant relationship is not nurturing as it does not facilitate the patient's health literacy and sense of control but rather encourages a focus on symptoms, procedures, pharmaceutical treatment, detrimental to their wellbeing.

### Conduct in Rigid Container-Not Contained Mode

Three participants experienced an authoritative attitude of providers toward them, i.e., those providers know best what they need and want, rather than empowering them. Disempowerment was conscious. Participants reported that their views were unheard. Providers were perceived as technical, alienated, uncaring. Lack of provider presence made participants feel that the providers do not see them; they felt objectified, vulnerable, and powerless (4). They felt marginalized, and their self-value depreciated, exacerbating their anxiety and distress. To our surprise, six of the participants who experienced a rigid container-contained mode, expressed empathy toward providers and justified their misconduct.

### Conduct in an Inverted Container-Not Contained Mode

Participants with poor experiences identified with the provider despite the poor experiences of lack of bedside manner, incompetency, and apathy toward participants and their basic needs. We named this paradoxical phenomenon, 'An Inverted Container', extending the three modes of container-contained to a fourth mode, representing the patient's defense mechanism in lengthy acute care hospitalizations. Our explanation of the inverted container phenomenon draws on the Stockholm syndrome (40, 41). The Stockholm syndrome refers to a paradoxical psychological phenomenon (based on an internal contradiction), when people who are held captive express appreciation, praise, and positive feelings toward their captors. These positive expressions seem irrational considering the captives' poor experiences but are viewed as a response typical under emotional pressure, depression, fear, anxiety, leading the captive to mistakenly interpret lack of abuse as a kind-hearted gesture (42, 43). This response provides the captive an artificial sense of safety (44). We view the inverted container as a mode in which the patient contains the provider, rather than the provider serving as a container for the patient. We view the inverted container as a specific response of dependent patients to their negative experiences with providers in relationships of asymmetrical power. We present the inverted container as a behavioral response to traumatic experiences.

Previous studies described traumatic experiences of patients in crisis due to progressive illness and misconduct of professionals in lengthy hospitalizations (4, 20, 37). The context often dictates reactions and in hospitalization, positive patient experiences may lead to trust, gratitude, adherence, and resilience. Negative experiences may lead to distrust, anger, anxiety, lack of adherence and refusal to return to the hospital in the future (4). Yet, patients with traumatic hospitalization experiences may act as inverted containers, paradoxically justifying their providers' misconduct and framing it positively, not only during hospitalization but also after discharge. Justifications entailed empathy, understanding, and discounting the aggravation and the depreciation of self-worth caused by providers. Participants attributed the poor conduct of providers to their burnout, overload, stress, lack of training, and organizational culture.

### Container Modes and PCC

Only the active container-contained mode showed nurturing provider–patient interactions with positive attitudes toward patients. Such interactions demonstrate PCC and positively impact patients' outcomes and wellbeing (20). Supporting previous studies, participants experienced comfort through humanized care and empowering relationships with providers (45). In relationships of nurturing and comfort, patients' trust in the providers leads to improved medication-adherence and self-management of disease post-discharge [(36, 39); Guba, 2020; 2020b]. It should be noted, however, that the god-like image of providers interferes with the formation of a container that allows person-to-person closeness with the patient. Inverted containers are dangerous due to patients' avoidance of reporting their pain and physical distress, making it difficult to diagnose and treat the disease appropriately. Furthermore, inverted containers are dangerous since they foster distrust of providers and hospitals with grave consequences post-discharge that contradict PCC. Moreover, the phenomenon of inverted containers raises concerns regarding the capacity of hospitals and professionals to provide PCC.

Since 2013, Israeli hospitals report embracing patient-centeredness, but this study suggests that providers representing two out of four containers undermine PCC. This understanding emphasizes the challenge of implementing PCC in acute care. This study supports previous studies that documented dynamics of asymmetrical power between providers and patients in the provider-patient interaction (11, 46). Providers adopting the modes of rigid container, wall-free container-contained, and the inverted container, did not recognize patients' distress signals (11). Furthermore, providers who interacted through modes of rigid container, the wall-free container mode, and the inverted container, practiced power asymmetry in their interactions with participants, and communicated in a manner that fell short of PCC and even contradicted it (11). Moreover, echoing previous studies, some providers even presented clinical information to patients in a way that elicited anxiety and fear (47). Other providers, although aware of their power, spoke over the patient's head rather than humanized themselves (46). Our findings reveal a critical disconnect between hospitals' desire to provide PCC and practicing its tenets.

Hospitals focus on outcomes and relate to efficiency and effectiveness. The highly charged environment of acute care places a priority on clinical competence. Yet patients' psychological and emotional needs are also in critical need of attention at the time of their crisis (48). The capacity to attend to patients' psychological as well as physical needs, is essential to PCC. Caring is an essential prerequisite of balancing life-saving interventions with psychosocial care. Thus, although providers work in a time-constrained work environment, with limited resources and a hectic pace, their attitudes rather than resources are key to forming nurturing provider-patient interactions. Furthermore, without nurturing relationship meaningfulness in providers, their job-dissatisfaction and burnout will deepen (49). Prioritizing care to meet patient's expectations will enhance both the short-term value and the long-term value of providers' work (49).

To effectively provide PCC, provider-patient interaction in lengthy hospitalizations must move along two axes: a clinical axis and a relationship axis. Patients in repeated re-admissions due to progressive disease or acute conditions experience a crisis (4, 12). Shifting as required between the clinical axis and the relationship axis, a provider may contain the patient at their time of crisis, alleviate anxiety, build patient trust, and form a partnership that enables involvement, higher health literacy and post-discharge adherence. In the provider-patient interaction, processes of reflection, projection, transference take place just as they do in the clinic (18, 19, 50). When the provider listens to the patient, reflects the patient's experience, and acknowledges the patient's crisis, the provider is actively containing the patient; transformation takes place, enabling the patient to better process the traumatic experience of the body's betrayal and promote healing (12, 37, 51). Active listening and clarification enable this transformation to take place through intrapersonal processes so that the patient can change. Without active listening the patient is not contained.

Eigen (52) emphasizes that providers need to be sensitive to challenges and constraints rather inhibit their ability to nurture patients. He calls upon providers to integrate “being” with “doing”. His writing is dedicated to all those seeking to turn the sacred therapeutic space into a place of ”being“ despite burnout and a broken soul. Providers will be able to nurture, support, and collaborate with patients and other providers if they adopt the active container-contained mode in the complex relationships with patients. Only then will patients be better able to process their trauma. The provider is called upon to navigate the patient through this processing, over and over, to slowly create an experience of wellbeing, growth, and resilience. Devoted providers go beyond subjectivity and inter-subjectivity, to the primacy of being and experiencing, as a foundational condition of their existence and mission.

Providers work under increasing physical and mental stressful conditions when exposed to patients, and the physical and mental strain causes burnout resulting in psychological distance called Compassion Fatigue (49, 53–55). Compassion fatigue is manifested in the provider's inability or reduced ability to feel and express empathy and support for patients, limiting the capacity for implementing PCC (53). Previous studies indicated that 25% of providers show signs of compassion fatigue and are even characterized as being post-traumatic (56). While the patient's distress is key in assessing the quality of care, the therapist's distress is scarcely mentioned in the literature.

Fleming (57) discusses the mental pain experienced by therapists in their daily work. She asks how the therapist deals with a patient who evokes suffering and how the therapist's tolerance for their own mental suffering affects the success of the therapeutic relationship. Following Fleming, we raise the question of how providers lacking inner resources due to burnout and compassion fatigue treat patients who expose them to damage to their inner self? At the same time, Fleming recognizes mental pain as inevitable in work with a patient. To enable change, providers must accept, feel, and bear the mental pain that arises in the countertransference and therefore must strengthen their mental transformation skills. Providers must go beyond observation and interpretation, as major players in the recuperating process- to interact with the patient and deal with projective identification that turns the provider-patient dyad into one unit that constitutes change (58, 59).

The transformation that takes place in the provider, according to Bion (18), depends on the flexibility of providers' capacity for containment. In other words, the ability to receive and carry the patient's contents as well as to use them for the therapeutic process depends not only on clinical competency but also on the ability to investigate countertransference reactions among providers accompanied by mental pain. Based on theory and clinical experience, Fleming (57) suggests that mental pain is an inherent part of the daily medical work. The clinical example that Fleming gives illustrates the consequences of the provider's mental pain on the course of treatment of the patient and its importance for working properly to be attentive to patients. The threshold of mental pain that provider are willing to bear as part of their profession, and the strategies they develop for dealing with the mental pain caused by the provider-patient interaction, are important issues for training and professional support.

### Practice Implication

PCC is resource-intensive and can only occur within supportive medical systems (60). Providers require ongoing capacity-building, adequate resources in terms of redesigning how care is offered, incentive schemes, as well as active assessment and feedback (61). Providers work in complex, fast-paced environments where competing clinical priorities and growing patient rosters make basic quality care, let alone PCC, difficult to achieve (11, 62). Without adequate organizational support, providers desiring to deliver PCC may lack a clear understanding of how to balance competing demands (63). Interventions to implement PCC are a). Work with providers to acknowledge their power and its relevance within the patient interactions in acute care. b). Guide providers to routinely reflect on patient-provider power dynamics (46). C). Support providers with on-going education and professional development on PCC, with explicit training about how to contain patients in delivery of care. d). Expose providers to the Treatment Escalation Plans to explain conditions, share decisions with patients whose health is deteriorating (64). e). Since reducing gaps in training on PCC communication is challenging given the high stress and burnout of providers, hospitals are called upon to focus on “person-centered care,” as a prerequisite to PCC, and practice policies that mitigate providers' stress. f). Last, modifying the term PCC to “person-centered care” may shape a perception of patients as people with needs beyond the clinical perspective (61).

Training should be directed at (a) Shaping the caring virtue. (b) Reflecting on providers' convictions about the needs and interpretations of interactions with providers. (c) Discuss what it means for patients “to be comfortable”. (d) Ways to incorporate insights into practice, e.g., interventions to optimize patient's comfort. Managements are called upon to establish mechanisms to contain the distress, suffering and grief of providers, enabling them to serve as containers for patients (48). Continuing education for providers may focus on the art of Doing and Being and on integrating empathy and compassion into practice by analysis of container modes in patients' narratives.

## Limitations

The emergence of the novel mode of inverted container may be, as in any qualitative study, dependent on socio-cultural characteristics of providers and raises the question regarding existent modes of containers in acute care in other countries between providers and patients who depend on their providers to fulfil their emotional needs. Also, because emotionally processing the hospitalization is a multi-phase temporal process, the times of conducting the interviews may have shaped the choice of the narrative. Last, the differences in age among participants may shape differences in patient expectations of being contained.

## Directions for Future Studies

Future qualitative studies may validate the containing modes identified in this study with a large sample and add an interview during the hospitalization period. Future empirical quantitative studies are called upon to examine the prevalence of each container mode in provider-patient interactions in lengthy hospitalizations. We also suggest that quantitative studies will testing the prevalence of the modes of containing by attributes conceptualized in this study. These future studies may be essential for developing training and continued education to attain PCC. As for providers, we propose exploring providers' perspective on their identity and foci, as providing clinical care, or clinical care and emotional support. Modes of containing patients may be explored as well as barriers to use the active container-contained mode in delivery of care. Such insights will promote continued education to promote PCC.

## Data Availability Statement

The original contributions presented in the study are included in the article/supplementary material, further inquiries can be directed to the corresponding author/s.

## Ethics Statement

The studies involving human participants were reviewed and approved by the Ethics Board of the College of Management Academic Studies. The patients/participants provided their written informed consent to participate in this study.

## Author Contributions

GG: conceptualization, methodology, data collection, data analysis, writing the original draft, and review and editing. SB-A: data analysis and review. Both authors contributed to the article and approved the submitted version.

## Conflict of Interest

The authors declare that the research was conducted in the absence of any commercial or financial relationships that could be construed as a potential conflict of interest.

## Publisher's Note

All claims expressed in this article are solely those of the authors and do not necessarily represent those of their affiliated organizations, or those of the publisher, the editors and the reviewers. Any product that may be evaluated in this article, or claim that may be made by its manufacturer, is not guaranteed or endorsed by the publisher.

## Appendix

### Exhibit A: The Broadcast-Translation Function and Identification Mechanisms

The mother transforms the messages the baby conveys to her into actions that optimize the baby's chances of developing physical and mental health. When the mother is emotionally unavailable, anxious, or confused, she may leave the baby's distress untranslated or translate signals into confusion. The container is active rather than passive, as often mistakenly described. An active container has a capacity to contain searching, asking, examining, and debating. Containing capacity involves creativity, which is made possible when a mother is free to feel and contain the baby within her. The mother, functioning as a suitable container for the baby's needs, can turn hunger into satisfaction, pain into pleasure, loneliness into togetherness, and fear of death into serenity.

Bion (18, 19) based the container-container function on Melanie Klein's projective identification mechanism, whereby discomfort resulting from hunger, fatigue, pain, etc., creates frustration and difficult, unprocessed experiences for the baby. (59). The baby throws the frustration at the parent, while the parent tries to take in and process (termed “reverie”) what provoked the baby's frustration response and provides the baby with a response. With time and accumulation of similar experiences, the parent's processing action resonates again and again in the baby, until the baby begins to recreate it on its own, thus forming a container-container relationship. When the mother suffers from the inability to reverie, to process the frustrations and fears of the baby, she does not function as an optimal container. The object, who is expected to mitigate, filter, and process the threat and fears, is perceived as a poor object. The mother reacts, at times, aggressively to the experiences of the baby, without offering internal processing and containment, and leaving the baby exposed to boundless fear.

## References

[1] Institute of Medicine (2020). Crossing the Quality Chasm: A New Health System for the 21st Century. Washington, DC: National Academies Press. 25057539

[2] Manzer JL Bell AV. The limitations of patient-centered care: The case of early long-acting reversible contraception (LARC) removal. Soc Sci Med. (2022) 292:114632. 10.1016/j.socscimed.2021.114632 34891032

[3] Institute of Medicine (2020). Envisioning the National Health Care Quality Report. Washington, DC: National Academies Press. 25057551

[4] Gabay G. Patient self-worth and communication barriers to Trust of Israeli Patients in acute-care physicians at public general hospitals. Qual Health Res. (2019) 29:1954–66. 10.1177/1049732319844999 31043144

[5] Tomaselli G Buttigieg SC Rosano A Cassar M Grima G. Person-centered care from a relational ethics perspective for the delivery of high quality and safe healthcare: a scoping review. Front Public Health. (2020) 8:44. 10.3389/fpubh.2020.00044 32211362 PMC7067745

[6] Shaller D. Patient-centered care: what does it take?. New York, NY: Commonwealth Fund. (2007). p. 1–26. https://www.commonwealthfund.org/publications/fund-reports/2007/oct/patient-centered-care-what-does-it-take (accessed October 20, 2020).

[7] Agency for Healthcare Research and Quality. National Healthcare Quality Report. Publication No. 06-0018, Rockville, MD: AHRQ, (2005).

[8] Planetree. (2020). Available online at: www.planetree.org (accessed Feburary 10, 2020).

[9] Fenton AT Elliott MN Schwebel DC Berkowitz Z Liddon NC Tortolero SR. Unequal interactions: Examining the role of patient-centered care in reducing inequitable diffusion of a medical innovation, the human papillomavirus (HPV) vaccine. Soc Sci Med. (2018) 200:238–48. 10.1016/j.socscimed.2017.09.030 29157686 PMC6413315

[10] O'Brien R Beeke S Pilnick A Goldberg SE Harwood RH. When people living with dementia say ‘no': Negotiating refusal in the acute hospital setting. Soc Sci Med. (2020) 263:113188. 10.1016/j.socscimed.2020.113188 32823045

[11] Dubbin LA Chang JS Shim JK. Cultural health capital and the interactional dynamics of patient-centered care. Soc Sci Med. (2013) 93:113–20. 10.1016/j.socscimed.2013.06.014 23906128 PMC3887515

[12] Gabay G. In the quest of resilience in elder patients: Solutogenics. In: Handbook of Ethnography in Healthcare Research. Routledge (2020). p. 301–12.

[13] Shim JK. Cultural health capital: a theoretical approach to understanding health care interactions and the dynamics of unequal treatment. J health Soc Behav. (2010) 51:1–15. 10.1177/0022146509361185 20420291 PMC10658877

[14] Institute for Patient- Family-Centered Care. (2020). Available online at: http://www.familycenteredcare.org/ (accessed Feburary 10, 2020).

[15] Luxford K Safran DG Delbanco T. Promoting patient-centered care: a qualitative study of facilitators and barriers in healthcare organizations with a reputation for improving the patient experience. Int J Qual Health Care. (2011) 23:510–5. 10.1093/intqhc/mzr024 21586433

[16] Circenis K Millere I. Compassion fatigue. burnout and contributory factors among nurses in Latvia. Procedia Soc Behav Sci. (2011) 30:2042–6. 10.1016/j.sbspro.2011.10.395

[17] Clouston SA Natale G Link BG. Socioeconomic inequalities in the spread of coronavirus-19 in the United States: A examination of the emergence of social inequalities. Soc Sci Med. (2021) 268:113554. 10.1016/j.socscimed.2020.113554 33308911 PMC7703549

[18] Bion WR. A theory of thinking. Int J Psychoanal. (1962) 43:306–10. 13968380

[19] Bion RWR. Second *Thoughts*. London: Heineman. (1967).

[20] Gabay G. From the crisis in acute care to post-discharge resilience–The communication experience of Geriatric patients: a qualitative study. Scand J Caring Sci. (2021) 35:123–33. 10.1111/scs.12826 32068292

[21] Ogden T. On the dialectical structure of experience. Contemp Psychoanal. (1988) 23:17–45. 10.1080/00107530.1988.10746217

[22] Winnicot DW. The Maturational Process and the Facilitating Environment. London: Routledge. (1986).

[23] Billow RM. Relational levels of the “container-contained” in group therapy. Group (2000) 24:243–59. 10.1023/A:1026603924963

[24] Bion WR. Eine theorie des denkens. Psyche. (1963) 17:426–35.

[25] Guba EG Lincoln YS. Competing paradigms in qualitative research. In: N. K. Denzin, N.K. and Lincoln, Y. S. (eds.), Handbook of Qualitative Research. Newbury Park, CA: Sage. (1994). p. 105–117.

[26] Malterud K Siersma VD Guassora AD. Sample size in qualitative interview studies: guided by information power. Qual Health Res. (2016) 26:1753–60. 10.1177/1049732315617444 26613970

[27] Morse JM. Ethics in action: Ethical principles for doing qualitative health research. Qual Health Res. (2007) 17:1003–5. 10.1177/1049732307308197 17928474

[28] Josselson R. Interviewing For Qualitative Inquiry: A Relational Approach. New York: Guilford Press. (2013).

[29] Kumar S Cavallaro L. Researcher self-care in emotionally demanding research: A proposed conceptual framework. Qual Health Res. (2018) 28:648–58. 10.1177/1049732317746377 29224510

[30] Hunt MR. Strengths and challenges in the use of interpretive description: reflections arising from a study of the moral experience of health professionals in humanitarian work. Qual Health Res. (2009) 19:1284–92. 10.1177/1049732309344612 19690208

[31] Segel S. (2009). Can universal healthcare work? A look at Israel's successful model. Physicians News Digest. Available online at: https://physiciansnews.com/2009/10/01/can-universal-healthcare-work-a-look-at-israels-successful-model/ (accessed March 10, 2022).

[32] Clarfield AM Manor O Nun GB Shvarts S Azzam ZS Afek A. Health and health care in Israel: an introduction. Lancet. (2017) 389:2503–13. 10.1016/S0140-6736(17)30636-0 28495109

[33] Crisp N Chen L. Global supply of health professionals. N Engl J Med. (2014) 370, 2247–8. 10.1056/NEJMc1404326 24897096

[34] Hewitt J. Ethical components of researcher—researched relationships in qualitative interviewing. Qual Health Res. (2007) 17:1149–59. 10.1177/1049732307308305 17928485

[35] Saldaña J. The Coding Manual for Qualitative Researchers. Thousand Oaks, CA: Sage. (2021).

[36] Gabay G. Exploring perceived control and self-rated health in re-admissions among younger adults: a retrospective study. Patient Educ Couns. (2016) 99:800–6. 10.1016/j.pec.2015.11.011 26626068

[37] Gabay G. A nonheroic cancer narrative: Body deterioration, grief, disenfranchised grief, and growth. OMEGA-Journal of Death and Dying. (2021) 83:287–309. 10.1177/0030222819852836 31138010

[38] Moyle W. Nurse–patient relationship: A dichotomy of expectations. Int J Ment Health Nurs. (2003) 12:103–9. 10.1046/j.1440-0979.2003.00276.x 12956021

[39] Gabay G. Perceived control over health, communication and patient–physician trust. Patient Educ Couns. (2015) 98:1550–7. 10.1016/j.pec.2015.06.019 26187177

[40] Cantor C Price J. Traumatic entrapment, appeasement and complex post-traumatic stress disorder: evolutionary perspectives of hostage reactions, domestic abuse and the Stockholm syndrome. Aust N Z J Psychiatry. (2007) 41:377–84. 10.1080/00048670701261178 17464728

[41] Jülich S. Stockholm syndrome and child sexual abuse. J Child Sex Abuse. (2005) 14:107–129. 10.1300/J070v14n03_06 16203697

[42] Logan MH. Stockholm syndrome: Held hostage by the one you love. Violence Gender. (2018) 5:67–9. 10.1089/vio.2017.0076

[43] Price JS Gardner Jr R Erickson M. Can depression, anxiety and somatization be understood as appeasement displays? J Affect Disord. (2004) 79:1–11. 10.1016/S0165-0327(02)00452-4 15023475

[44] Woody S Rachman S. Generalized anxiety disorder (GAD) as an unsuccessful search for safety. Clin Psychol Rev. (1994) 14:743–53. 10.1016/0272-7358(94)90040-X

[45] Coelho A Parola V Escobar-Bravo M Apóstolo J. Comfort experience in palliative care: a phenomenological study. BMC Palliat Care. (2016) 15:71. 10.1186/s12904-016-0145-0 27484497 PMC4971655

[46] Nimmon L Stenfors-Hayes T. The “handling” of power in the physician-patient encounter: perceptions from experienced physicians. BMC Med Educ. (2016) 16:1–9. 10.1186/s12909-016-0634-0 27091146 PMC4835893

[47] Henderson L. The Compassionate-Mind Guide to Building Social Confidence: Using Compassion-Focused Therapy to Overcome Shyness and Social Anxiety. Oakland, CA: New Harbinger. (2011).

[48] Gabay G Asher SB. From a View of the Hospital as a System to a View of the Suffering Patient. Front Public Health. (2021) 9:2150. 10.3389/fpubh.2021.800603 35071174 PMC8782256

[49] Gabay G Netzer D Elhayany A. Shared trust of resident physicians in top-management and professional burnout: A cross-sectional study towards capacity for patient-focused care, peer support and job expectations. Int J Health Plann Manage. (2022) 1–15. 10.1002/hpm.3479 35426169

[50] Winnicot DW. Ego Distortions in Terms of True Self and False Self. The Maturational Processes and the Facilitating Environment. (1965).

[51] Quinodos D. The psychoanalytic setting as the instrument of the container function. Int J Psychoanal. (1992) 73:627–39. 1483843

[52] Eigen M. The Sensitive Self. Middletown, CT: Wesleyan University Press. (2004).

[53] Figley CR. Compassion fatigue: Psychotherapists' chronic lack of self-care. J Clin Psychol. (2002) 58:1433–41. 10.1002/jclp.10090 12412153

[54] Kwak H McNeeley S Kim SH. Emotional labor, role characteristics, and police officer burnout in South Korea: The mediating effect of emotional dissonance. Police Quarterly. (2018) 21:223–49. 10.1177/1098611118757230

[55] Yoder JA Litman GW. The phylogenetic origins of natural killer receptors and recognition: relationships, possibilities, and realities. Immunogenetics (2011) 63:123–41. 10.1007/s00251-010-0506-4 21191578 PMC3691731

[56] Sprang G Craig CD. Compassion satisfaction, compassion fatigue, and burnout in a national sample of trauma treatment therapists. Anxiety Stress Coping. (2010) 23:319–39. 10.1080/10615800903085818 19590994

[57] Fleming M. The mental pain of the psychoanalyst: a personal view. Int Forum Psychoanaly. (2005) 14:69–75. 10.1080/08037060510028181

[58] Malin A Grotstein JS. Projective identification in the therapeutic process. Int J Psychoanal. (1966) 47:26–31. 5959732

[59] Mawson C. Bion, Freud, and Klein: Interpretation as Freud's specific Action and Bion's Container. Int J Psychoanaly. (2016) 30:1–20. 10.1111/1745-8315.12668 28542871

[60] Epstein RM Street RL. The values and value of patient-centered care. Ann Fam Med. (2011) 9:100–3. 10.1370/afm.1239 21403134 PMC3056855

[61] Eklund JH Holmström IK Kumlin T Kaminsky E Skoglund K Höglander J. “Same same or different?” A review of reviews of person-centered and patient-centered care. Patient Educ Couns. (2019) 102:3–11. 10.1016/j.pec.2018.08.029 30201221

[62] Brandi K Fuentes L. The history of tiered-effectiveness contraceptive counseling and the importance of patient-centered family planning care. Am J Obstet Gynecol. (2020) 22:S873–S877. 10.1016/j.ajog.2019.11.1271 31794724

[63] Yphantides N Escoboza S Macchione N. Leadership in public health: new competencies for the future. Front Public Health. (2015) 3:24. 10.3389/fpubh.2015.00024 25767792 PMC4341427

[64] May C Myall M Lund S Campling N Bogle S Dace S. Managing patient preferences and clinical responses in acute pathophysiological deterioration: What do clinicians think treatment escalation plans do? Soc Sci Med. (2020) 258:113143. 10.1016/j.socscimed.2020.113143 32599414 PMC7369631

